# Effect of centchroman coadministration on the pharmacokinetics of metformin in rats

**DOI:** 10.4103/0253-7613.66836

**Published:** 2010-06

**Authors:** Jawahar Lal, Girish K. Jain

**Affiliations:** Pharmacokinetics & Metabolism Division, Central Drug Research Institute, CSIR, Lucknow - 226 001, India

**Keywords:** Centchroman, drug interaction, metformin, oral antidiabetic, oral contraceptive

## Abstract

**Objectives:**

To study the effect of centchroman, a non-steroidal oral contraceptive, coadministration on the pharmacokinetics of metformin in rats.

**Materials and Methods:**

The pharmacokinetic interaction of metformin was studied in normal Sprague-Dawley female rats with and without centchroman coadministration. Blood samples were analyzed using a validated high-performance liquid chromatography method to generate the pharmacokinetic profile of metformin. The C_max_ and t_max_ were directly read from the concentration–time data. Other pharmacokinetic parameters were estimated using non-compartmental analyses.

**Results:**

Metformin was monitored up to 10 h, and it exhibited a double-peak phenomenon. The C_max 1_, 2.62 ± 0.32 μg/ml, and C_max 2,_ 2.96 ± 0.65 μg/ml, occurred after 0.75 and 3 h post-dose, respectively. The mean residence time (MRT), AUC_0-4 h_ and volume of distribution (Vd/F) were 4.20 ± 0.30 h, 8.53 ± 1.89 μg.h/ml and 14.24 ± 5.42 L/kg, respectively. Following centchroman coadministration, metformin showed significantly (*P* < 0.05) higher C_max_ (C_max 1,_ 3.96 ± 0.55 μg/ml and C_max 2,_ 5.21 ± 0.59 μg/ml), AUC_0-4 h_ (12.28 ± 0.73 μg.h/ml) and Vd/F (18.29 ± 1.19 L/kg), but lower MRT (3.19 ± 0.36 h) than the values obtained after metformin dosing alone. However, AUC_0-t_ (17.74 ± 5.58 μg.h/ml) and clearance (3.76 ± 0.80 L/h/kg) remained unchanged.

**Conclusions:**

The results indicate that centchroman coadministration increases the rate but not the extent of absorption of metformin in rats. However, it does not seem to alter the pharmacokinetics of metformin to clinically significant levels.

## Introduction

Metformin has been used in the clinical management of type 2 diabetes mellitus and acts principally by improving insulin sensitivity of the peripheral tissue (chiefly skeletal muscle) and the liver, thus opposing insulin resistance. All oral antidiabetic drugs have the potential to interact with other medications, including oral contraceptive hormones, and if the result is hypoglycemia or hyperglycemia, the consequences can be serious.[1,2] DL-Centchroman is a non-steroidal once-a-week oral contraceptive, which provides pregnancy protection, and is administered in post-coital and weekly regimens. It is effective in the 30- and 60-mg once-a-week post-coital dose regimens. Because of its potent antiestrogenic and weak estrogenic activities, it is also effective against advanced breast cancer.[3–5] Drug interaction of centchroman and oral antidiabetic drug (metformin, glibenclamide and pioglitazone) coadministration in Sprague-Dawley female rats on days 1 through 5 post-coitum has shown significantly lower C_max_, t_max_ and AUC_0-24 h_ values of centchroman, but the clearance and volume of distribution remained unchanged. However, the AUC_0-t_ of centchroman remained unchanged with the coadministration of glibenclamide.[6] Therefore, it was deemed necessary to study the effect of centchroman coadministration on the pharmacokinetics of metformin to evaluate the possible drug interaction.

## Materials and Methods

### Chemicals

Pure reference standard of metformin was obtained from Wallace Pharmaceutical Ltd., Goa, India. Centchroman (purity >99%) was obtained from the Medicinal Chemistry Division, CDRI, Lucknow, India. High-performance liquid chromatography (HPLC)-grade acetonitrile and methanol were obtained from E Merck (India) Ltd., Mumbai, India. Ammonium acetate (AR grade), chloroform (HPLC-grade) and hydrochloric acid (LR grade) were obtained from Qualigens Fine Chemicals, Mumbai, India. Purified de-ionized water was obtained from a Milli-Q (Millipore, Bedford, MA, USA) water purification system. A 0.22-*μ*m cellulose membrane (Whatman International Ltd., Mailstone, England) was used for the filtration of buffer. Heparin sodium injection 25000 IU was obtained from Biological E. Limited, Hyderabad, India. Parafilm (Parafilm “M” Laboratory Film; American Can Company, Greenwich, CT, USA) was used for sealing the tubes.

Drug-free rat plasma was collected from healthy female Sprague-Dawley rats provided by the Laboratory Animal Services Division, CDRI, Lucknow, India and pooled to generate the drug-free pool of the biomatrix.

### Animals

Female Sprague-Dawley rats (200–280 g) were acclimatized in the animal room at least 1 day prior to the commencement of the study and were maintained on standard food and water *ad libitum*. The study protocols were prepared according to the guidelines specified by the local animal ethics committee. All experiments, euthanasia and disposal of carcasses were carried out as per the guidelines of the local animal ethics committee for animal experimentation.

### Preparation of the formulation

Rat dose of metformin was calculated from the standard clinical human dose on the basis of surface area [rat dose = {(human dose/average body weight}×7}].[7] 224.35 mg metformin.HCl (IP grade; ≡175 mg metformin) was dissolved in 5 ml triple-distilled water to obtain a strength of 35 mg/ml. Centchroman was prepared by dissolving 4.046 mg centchroman.HCl (≡3.75 mg centchroman) in 5 ml absolute alcohol-triple-distilled water mixture (25%, v/v) to obtain 0.75 mg/ml of centchroman. The total volume of centchroman and the coadministered drugs did not exceed 5 ml/kg in order to minimize the effect of hydrodynamics on drug absorption.

### Oral administration

Thirty rats were divided in two groups of 15 each. The overnight fasted rat received a single 70 mg/kg oral dose of metformin using a rat feeding needle and syringe. Two blood samples were withdrawn from each rat, one intracardiac puncture (~1.2 ml) and one from the inferior vena cava. Blood samples from the dosed rats were collected at 0.5, 0.75, 1, 1.5, 2, 3, 4, 6, 8 and 10 h using a heparinized syringe. Plasma was separated and the samples were stored at -50°C pending analysis. The same protocol was followed with the other group (15 rats) that received a single 70 mg/kg oral dose of metformin with a single 1.5 mg/kg oral dose of centchroman.

### Analytical procedures

The HPLC method was developed and validated to determine the concentration of metformin in rat plasma. Briefly, to each 0.2 ml of sample (blank, spiked or test) was added 20 *μ*L hydrochloric acid (1N) while vortexing followed by 0.6 ml acetonitrile and then vortexed for 1 min. Further, these samples were allowed to stand for 30 min and then centrifugated at 1000 g for 10 min. To the supernatant, transferred in a clean test tube, was added an equal volume of water and then vortexed. To this, 1 ml chloroform was added, vortexed for 30 s, sealed with parafilm and centrifugated at 1000 g for 10 min. Approximately 200 *μ*L supernatant was injected onto the HPLC system. The HPLC system was equipped with LC-10AD pumps with a SCL-10A VP system controller, 7125i Rheodyne injector fitted with a fixed 100 *μ*L loop, SPD-10A VP UV-VIS multiple wavelength detector set at 235 and Class-VP (version 6.12 SP2) software (Shimadzu, Japan) on personal computer. Separation was obtained on a Brownlee RP-18 column (5 *μ*m, 220 mm × 4.6 mm) coupled with a guard column (5 *μ*m, 30 mm × 4.6 mm) packed with the same material. Adequate numbers of QC samples were injected to ensure the acceptability of an analytical run. A calibration curve was constructed using response (peak height) against the respective concentration in calibration standards and the metformin concentration from rat plasma samples was read.

The HPLC method was validated in terms of reproducibility, recovery, accuracy and precision and then applied for estimation of metformin in rat plasma. Unknown concentrations of metformin were interpolated from the respective plasma standard curves drawn on each day of the sample analysis.

### Pharmacokinetic analysis

The peak plasma metformin concentration (C_max_ ) and its time of occurrence (t_max_ ) were directly read from the concentration–time data. Other pharmacokinetic parameters were determined on subjecting the concentration-time data to non-compartmental and compartmental analysis using WinNonlin (version 5.1) software. AUC was calculated using the Bailer method by the trapezoidal rule.[8]

### Statistical analysis

Student’s *t*-test was performed to test the effect of centchroman coadministration on the pharmacokinetics of metformin, and *P* <0.05 was considered to be significant.

## Results

The extraction procedure and the chromatographic conditions yielded a clean chromatogram for metformin. Recoveries of metformin from the spiked plasma samples (QC samples), calculated at low (100 ng/ml), medium (1000 ng/ml) and high concentrations (5000 ng/ml), ranged from 96.5% to 102%, with a coefficient of variation (CV) of <5% and a limit of quantitation of 100 ng/ml. Analysis of the QC samples at three concentration levels for three different days showed that the variation in the observed concentration (%bias) was -8.4 to 5.3%, whereas it varied between -14.1 and 4.8%. Percent relative standard deviation of the QC samples assayed on day 1 ranged from 6.6 to 12.9%, whereas it was 1.1 to 20% over a period of 3 days [Table 1].

**Table 1:** Intra- and interassay accuracy and precision of the HPLC method for the determination of metformin concentration

*Analysis of spiked sample*	*Low*	*Medium*	*High*
Theoretical concentration (ng/ml)	100	1000	5000
Observed concentration (mean ± SD)	99.2 ± 15.0	909.0 ± 60.1	5112.4 ± 324.0
Bias_intraassay_ (%)	-4.3	-8.4	5.3
Bias_interassay_ (%)	-14.1	-9.2	4.8
RSD_intraassay_ (%)	12.9	7.6	6.6
RSD_interassay_ (%)	20	1.1	5.6

RSD- Relative standard deviation

The concentration–time profile and the pharmacokinetic parameters of metformin after a single 70 mg/kg oral dose of metformin with and without centchroman coadministration in Sprague-Dawley female rats is shown in Table 2. The rats administered with metformin dose alone showed two C_max_ of metformin (C_max 1_, range: 2.31–2.94; C_max 2_, range: 2.27–3.55 *μ*g/ml) that occurred after 0.75 and 3 h post-dose, respectively. It was monitored up to a period of 10 h. Because of the occurrence of a double-peak phenomenon, the data sets could not be fitted to an appropriate compartmental pharmacokinetic model. Hence, the pharmacokinetic parameters were determined by a non-compartmental analysis of the plasma concentration–time data using WinNonlin program (Ver 5.1), and are listed in Table 2. Moreover, the Bailer method[9] was applied to estimate the standard error of AUC in each treatment group. AUC_0-4 h_ and AUC_0-t_ were 8.53 ± 1.89 and 17.74 ± 1.68 *μ*g.h/ml, respectively. The mean residence time (MRT) varied from 3.89 to 4.48 h. Apparent volume of distribution (Vd/F) and clearance (Cl/F) averaged to 14.24 L/kg and 3.76 L/h/kg, respectively.

**Table 2:** Pharmacokinetic parameters of metformin with and without centchroman coadministration in female rats

*Parameters*	*Metformin (70 mg/kg)*	
	*Without centchroman coadministration*	*With centchroman coadministration (1.5 mg/kg)*
C_max, 1_ (μg/ml)	2.62 ± 0.32	3.96 ± 0.55*
C_max, 2_ (μg/ml)	2.96 ± 0.65	5.21 ± 0.59*
t_max, 1_ (h)	0.75	1.0
t_max, 2_ (h)	3	3
AUC_0-4 h_ (μg.h/ml)	8.53 ± 1.89	12.28 ± 0.73*
AUC_0-t_ (μg.h/ml)^a^	17.75 ± 1.68	16.78 ± 1.74
Vd/F (L/kg)	14.24 ± 5.42	18.29 ± 1.19
Cl/F (L/h/kg)	3.76 ± 0.80	3.98 ± 0.45
MRT (h)	4.20 ± 0.30	3.19 ± 0.36*

C_max_, plasma peak concentration; t_max_, time to peak concentration; AUC_0-4_, area under the concentration–time curve from time zero to 4 h; AUC_0-t_, area under the concentration–time curve from time zero to the last sampling time; Vd/F, volume of distribution; Cl/F, clearance; MRT, mean residence time; Values are expressed as mean ± SD;

a Bailer’s method for AUC variance;

* *P*<0.05

The Sprague-Dawley female rats treated with both metformin and centchroman also showed two C_max_ of metformin. With centchroman coadministration, the C_max_ of metformin were higher than that after metformin dosing alone, and ranged from 3.51 to 4.58 *μ*g/ml (C_max 1_) and 4.52 to 5.60 *μ*g/ml (C_max 2_). The C_max 1_ was delayed and occurred after 1.0 h post-dose, but C_max 2_ did not show any variation and occurred after 3 h post-dose. The AUC_0–4 h_ varied between 11.60 and 13.06 *μ*g.h/ml [Table 2]. Vd/F (range, 17.0–19.36 L/kg) was higher than that after metformin dosing alone. However, MRT (range, 2.93–3.60 h) was lower than that after metformin dosing alone. The Cl/F and AUC_0-t_ varied from 3.48 to 4.36 l/h/kg and 15.19 to 18.63 *μ*g.h/ml, respectively, and were similar to the values obtained after metformin dosing alone.

## Discussion

The assay method was validated before use. The limit of quantification (LOQ) in rat plasma was determined to be 100 ng/ml. A linear relationship existed between peak heights and concentrations of metformin (20–1000 ng/ml) in the mobile phase and plasma concentrations of metformin (100–5000 ng/ml), respectively. The recoveries of metformin were >90%, with a CV of <5%. Between and within-assay bias, inter- and intrabatch precision were within the acceptable limits at quality control (QC) samples (*n* = 3) at the low, medium and high concentration levels.[9]

Mean plasma concentration–time profiles indicated that the plasma metformin concentrations peaked (C_max 1_) slightly later than the metformin-alone treatment. However, C_max 2_ occurred at the same time (t_max 2_, 3 h), and both the C_max_ were significantly higher (*P* < 0.05) than that of the metformin-alone group. However, the difference in t_max 1_ was not statistically significant. Although the AUC_0-t_ did not show a significant variation after oral administration of both drugs, the AUC_0-4 h_ of metformin was significantly greater (*P* < 0.05) than that of the metformin-alone treatment Table 2. Moreover, the AUC_0-4 h_ /AUC_0-t_ ratio, which averaged to 0.49 after metformin-alone treatment, significantly increased (0.74 ± 0.10) after the oral administration of both drugs. AUC_0-4 h_ was used as one of the outcomes because that is the period where most of the differences can be observed between treatments with or without centchroman. The MRT, indicative of the elimination half-life, significantly decreased (*P* < 0.05) than the values obtained after metformin dose alone, indicating that the rate of elimination of metformin was significantly increased after oral administration of both drugs [Table 2].

In contrast to metformin, the C_max_, t_max_ and AUC_0-24 h_ values of centchroman were significantly lower in the presence of metformin after oral administration. Moreover, C_max 2_ and AUC of centchroman were decreased by 27% and 32%, respectively, in the presence of metformin and pioglitazone than in their absence. However, the AUC_0-t_ of centchroman remained unchanged with the co-administration of glibenclamide.[6] Furosemide, nifedipine and cimetidine have been found to increase the plasma metformin C_max_ and AUC.[10] In the present study, the total extent of absorption (AUC_0-t_) of metformin did not show variation as compared to metformin alone [Table 2]. Also, the Vd/F and Cl/F values of metformin did not vary after oral administration of both drugs. Therefore, changes in the pharmacokinetics of metformin could be regarded as a consequence of the altered initial absorption of metformin.

Double peaks have also been observed with other drugs, including acetaminophen, aspirin, cimetidine, flurbiprofen, furosemide and penicillamine. Several hypotheses based on region-dependent variation in absorption, enterohepatic recirculation, delayed or variable gastric emptying and intestinal transit rates and intestinal bacterial reconversion of biliary metabolite have been proposed to account for these observations.[11,12] In the present study, the double-peak phenomenon is not clear as metformin is primarily absorbed from the small intestine, excreted unchanged in the urine and does not undergo hepatic metabolism or biliary excretion.[13]

Lactic acidosis, a life-threatening condition characterized by low arterial pH (<7.35) and elevated arterial lactate levels (5.0 mEq/L in humans), is a well-recognized complication of biguanide therapy that is potentially serious.[1,14] Factors that decrease metformin excretion or increase blood lactate levels may cause lactic acidosis. Renal dysfunction will cause a significant increase in the plasma biguanide concentration, resulting in lactic acidosis. But, the development of lactic acidosis appears to be unrelated to plasma metformin concentrations.[15] Therefore, the increased plasma metformin concentration is unlikely to lead to lactic acidosis.

Metformin is negligibly bound to plasma proteins and is, therefore, less likely to interact with highly protein-bound drugs such as centchroman.[16] Metformin is eliminated by active secretion in the kidney and it is likely that one or more organic cation transporters are involved in this process.[17–19] Dresser *et al*.[20] demonstrated that there are compound-dependent differences in the specificities of hOCT1 and hOCT2 (these differences may contribute to the organ-specific elimination of the drugs) and observed that metformin interacts with hOCT1 and hOCT2. The kidney transporter, hOCT2, prefers smaller hydrophilic substrates in contrast to the liver transporter, hOCT1, which may interact with larger, more hydrophobic compounds. Centchroman is extremely lipophilic (logP, 7.04),[21] suggesting that hOCT1 and hOCT2 may be the molecular site of metformin centchroman drug–drug interaction. Therefore, it may be concluded that although the coadministration of centchroman significantly enhanced the initial oral absorption of metformin, other pharmacokinetic parameters remained unaltered and hence it may not be of any clinical significance.
